# Management of a Patient With Acute Abdomen and Thyroid Storm

**DOI:** 10.1016/j.aed.2025.04.010

**Published:** 2025-05-08

**Authors:** Sophie Eschlböck, Alexandros Lalos, Adisa Poljo, Beatrice Kern, Alberto Posabella, Stephanie Taha-Mehlitz

**Affiliations:** 1 Clarunis – University Digestive Health Care Center, St. Clara Hospital and University Hospital of Basel, Basel, Switzerland; 2 Faculty of Medicine, University of Basel, Basel, Switzerland

**Keywords:** thyroid storm, mesenteric ischemia, multidisciplinary management

## Abstract

**Background:**

Acute abdomen often requires urgent surgical intervention. In certain instances, thyrotoxicosis can contribute to the onset of acute abdomen. This relationship complicates both diagnosis and management. Therefore, understanding the interplay between thyrotoxicosis and acute abdomen is essential for implementing effective treatment strategies. This case report aims to illustrate the diagnostic and therapeutic challenges encountered in managing a patient with mesenteric ischemia due to thyroid storm, emphasizing the importance of a multidisciplinary approach in achieving favorable outcomes.

**Case Report:**

We describe a case of a 54-year-old female who presented with acute abdominal pain, rapidly deteriorating to respiratory failure requiring intubation. Initial investigations revealed an embolic occlusion of the superior mesenteric artery and signs of bowel ischemia as well as cardiopulmonary decompensation. To evaluate the cause of the cardiac failure, subsequent thyroid function tests confirmed hyperthyroidism. The patient underwent endovascular embolectomy, bowel resection, and later a total thyroidectomy.

**Discussion:**

The patient’s acute abdomen was managed with embolectomy and bowel resection. A Burch-Wartofsky score of 75 was highly suggestive of thyroid storm, leading to an urgent total thyroidectomy after interdisciplinary discussion. Histopathological examination confirmed chronic thyroid inflammation with increased endocrine activity.

**Conclusion:**

This case highlights the serious complication of mesenteric ischemia due to thyroid storm. Endovascular thrombectomy and bowel resection effectively managed the ischemia, while thyroidectomy facilitated a rapid return to a euthyroid state. This case serves as a valuable reference for the management of similar presentations, demonstrating the potential for successful outcomes with timely, aggressive treatment.

**Highlights**

• Thyroid storm can cause embolic events leading to mesenteric ischemia and acute abdomen
• Early recognition of thyrotoxicosis is crucial in patients presenting with acute embolic events
• Multidisciplinary management improves outcomes in thyroid storm-induced mesenteric ischemia
• This case underscores the need for thyroid function testing in unexplained embolic events

**Clinical Relevance**

This case emphasizes the importance of recognizing thyroid storm as a potential cause of mesenteric ischemia. Prompt diagnosis and a multidisciplinary approach can significantly improve patient outcomes by addressing both the acute abdomen and the endocrine dysfunction simultaneously.

## Introduction

Acute abdomen, typically characterized by severe and sudden abdominal pain, often requires urgent surgical intervention. In some cases, thyrotoxicosis can directly or indirectly contribute to the development of acute abdomen, complicating both diagnosis and management.^1^, ^2^, ^3^, ^4^ Recognizing this interplay is crucial for guiding appropriate treatment strategies. In this case report, we describe a patient who initially presented with acute abdominal symptoms, necessitating urgent endovascular and surgical intervention. It was only during the course of treatment that thyrotoxicosis was identified as the underlying precipitant.

## Case Report

A 54-year-old female with no significant medical history or regular medications presented initially to a peripheral hospital with severe acute abdominal pain and diarrhea. Upon admission her electrocardiogram showed tachycardic atrial fibrillation. The patient's condition rapidly deteriorated. She required intubation due to respiratory failure and was transferred to our center for further evaluation and treatment.

**Fig. 1:**
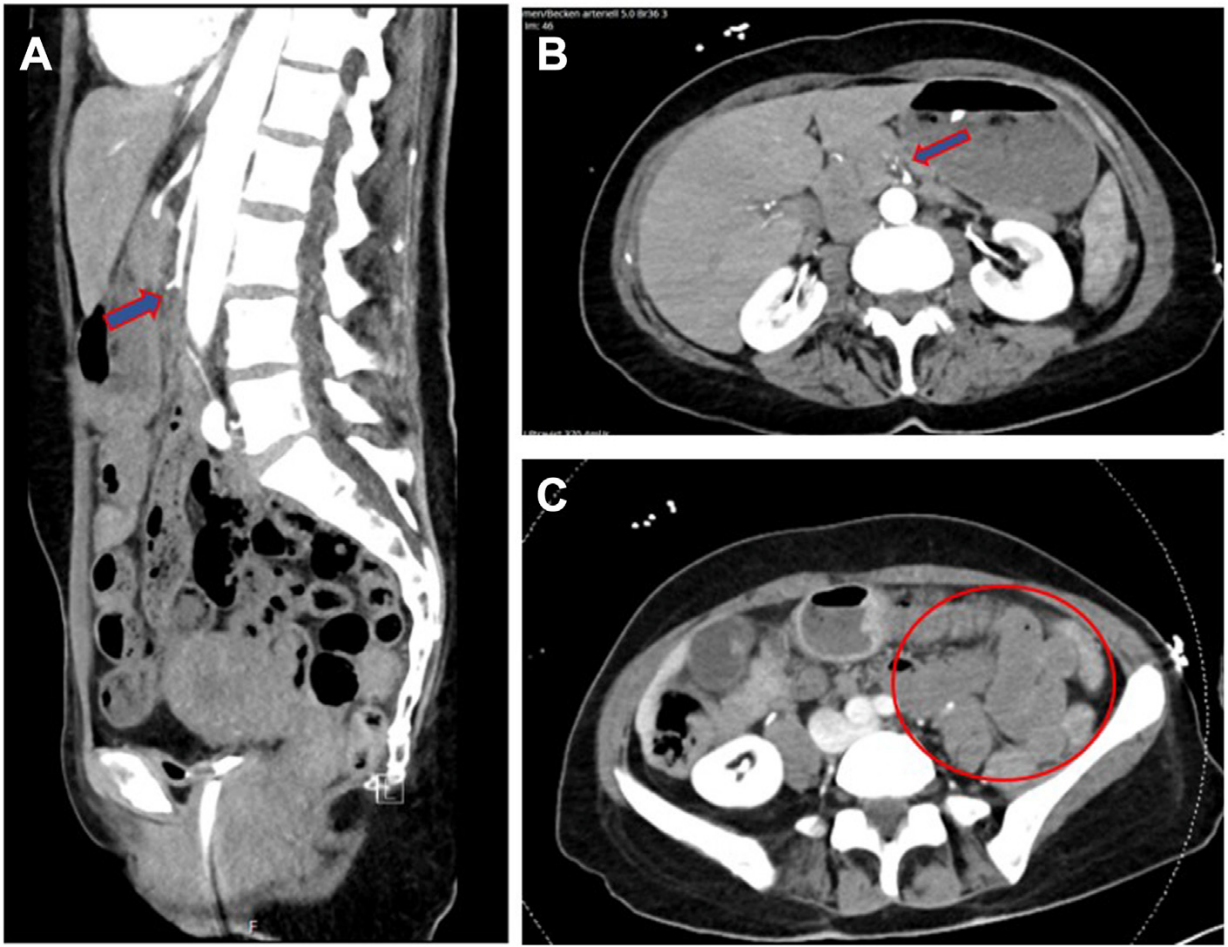
*A*, Sagittal view of the embolic occlusion of SMA approximately 4.5 cm distal to its origin from the aorta. *B*, Axial view of the embolic occlusion of SMA. *C*, Hypoenhancement of the small bowel wall indicating bowel ischemia.

The laboratory results at admission were largely unremarkable except for an elevated white blood cell count (26.1 G/L, reference range: 3.5–10 G/L) and an elevated level of Troponin T (209 ng/L, reference range: < 14 ng/L). Due to the acute abdominal presentation and unclear history, a computed tomography angiography of the thorax, abdomen, and pelvis was performed. The scan revealed an embolic occlusion of the superior mesenteric artery (SMA) approximately 4.5 cm distal to its origin from the aorta. Additionally, there were signs of bowel ischemia as well as cardiopulmonary decompensation characterized by interstitial and alveolar pulmonary edema (Fig. 1).

**Fig. 2:**
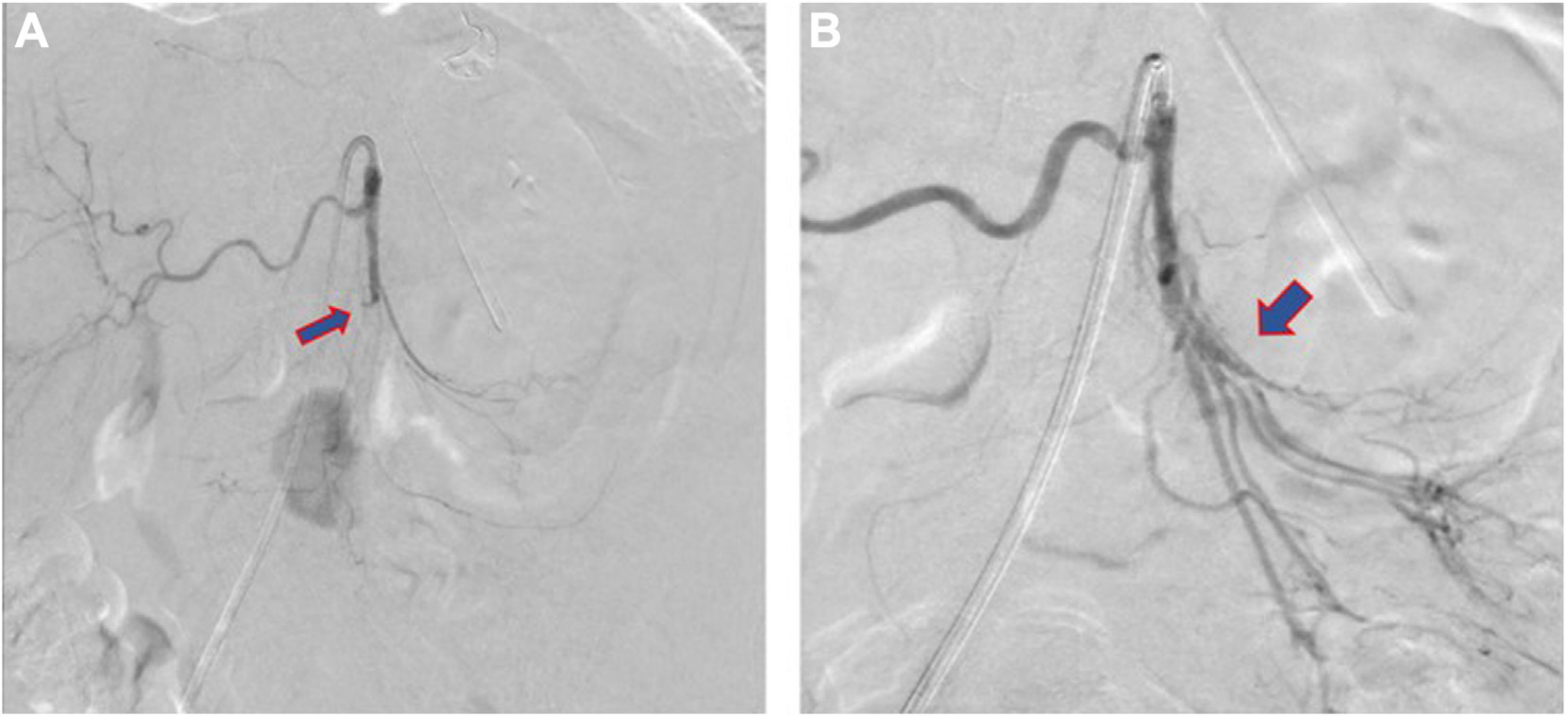
*A*, The DSA indicates a thrombotic occlusion of the SMA located approximately 4.5 cm from its origin. *B*, Recanalization of SMA after the embolectomy. *DSA* = digital subtraction angiography; *SMA* = superior mesenteric artery.

The first step in the treatment, performed by an interventional radiologist, was an embolectomy to achieve recanalization of the SMA (Fig. 2).

At the conclusion of the embolectomy, the patient was immediately taken to the operating theater, where a median laparotomy was performed to explore the abdomen. Necrotic small intestine was identified 140 cm distal to the Treitz ligament, and the right hemicolon also showed signs of necrosis. Consequently, a segmental resection of the small intestine and a right hemicolectomy was performed. The patient, who remained unstable and required a high dose of vasopressors, was then transferred to the intensive care unit for further monitoring and management. The histopathology report showed fresh hemorrhagic necrosis of the small and large intestines, as well as the appendix, consistent with ischemia.

Further diagnostic workup in the intensive care unit included transthoracic echocardiogram (TTE), which revealed severely impaired left and right ventricular function. A few hours later, a follow-up TTE performed by the cardiologist identified a round, mobile structure in the midventricular region of the inferolateral wall of the left ventricle. This finding raised the suspicion of a possible thrombus.

To further investigate the underlying causes of the acute cardiac failure, a comprehensive thyroid evaluation was conducted. The results displayed in Table 1 strongly indicate a state of hyperthyroidism, as evidenced by the significantly suppressed thyroid-stimulating hormone level, elevated free T4, and free T3 levels.

**Table 1:** Thyroid Parameters

Parameter	Value	Reference range
TSH, mIU/L	0.006	0.332-4.4490
Free T4, pmol/L	76.0	11.9-21.9
Free T3, pmol/L	30.9	2.6-5.6
T3, nmol/L	7.4	1.2-3.2
Anti-TPO-antibodies, kIU/L	389	<34
Anti-TSH-receptor-antibodies (TRAB), IU/L	20.8	<1.8

Abbreviations: Anti-TPO = anti-thyroid peroxidase antibodies; Free T3 = free triiodothyronine; Free T4 = free thyroxine; Total T3 = total triiodothyronine; TSH = thyroid-stimulating hormone.

Given the clinical presentation and the untreated hyperthyroidism, we calculated the Burch-Wartofsky Point Scale to assess the likelihood of a thyroid storm (Table 2). The score was 75, which is highly suggestive of a thyroid storm, indicating an urgent need for intervention and management of this critical condition. In view of the hemodynamic instability, 2 attempts at electrical cardioversion were made, although only a brief conversion to sinus rhythm could be achieved. Initially, treatment with Amiodarone was withheld due to the hyperthyroid metabolic state. The treatment with methimazole and hydrocortisone was unsuccessful with continued hemodynamically significant tachycardic atrial fibrillation. After an interdisciplinary discussion with the endocrinologists, an urgent total thyroidectomy was deemed necessary. The histopathology report indicated a tumor-free thyroid with chronic inflammation and signs of increased endocrine activity. Postoperatively, the patient rapidly became hemodynamically stable.

**Table 2:** Burch-Wartofsky Point Scale for Thyroid Storm

Category	Condition	Points
Temperature °F (°C)	<99 (37.2)	0
	***9-99.9 (37.2-37.7)***	***+5***
	100-100.9 (37.8-38.2)	+10
	101-101.9 (38.3-38.8)	+15
	102-102.9 (38.9-39.2)	+20
	103-103.9 (39.3-39.9)	+25
	≥104.0 (40.0)	+30
Central nervous system effects	Absent	0
	Mild (agitation)	+10
	***Moderate (delirium, psychosis, extreme lethargy)***	***+20***
	Severe (seizures, coma)	+30
Gastrointestinal-hepatic dysfunction	Absent	0
	***Moderate (diarrhea, nausea/vomiting, abdominal pain)***	***+10***
	Severe (unexplained jaundice)	+20
Heart rate (beats/minute)	<90	0
	90-109	+5
	110-119	+10
	120-129	+15
	130-139	+20
	***≥140***	***+25***
Congestive heart failure	Absent	0
	Mild (pedal edema)	+5
	Moderate (bibasilar rales)	+10
	***Severe (pulmonary edema)***	***+15***
Atrial fibrillation present	***No***	***0***
	Yes	+10
Precipitating event	***No***	***0***
	Yes	+10

A score of less than 25 is unlikely to represent a thyroid storm, 25-44 suggests an impending thyroid storm, and a score of 45 or more is highly suggestive of a thyroid storm. The findings specific to our patients are highlighted in italic and bold.

The initially identified floating structure in the left ventricle was no longer detectable in subsequent TTEs and transesophageal echocardiography after several days of therapeutic anticoagulation. Four days after the last operation, the patient was transferred to the surgical ward.

Throughout the hospitalization, the patient was comanaged by the nutritional counseling team, cardiologists, and endocrinologists. The patient was discharged in good condition to her home environment.

## Discussion

The case presented shows a dramatic example of the extreme effects of thyrotoxicosis, particularly in the context of thyroid storm, which is its rare and most severe manifestation. This condition presents as a hypermetabolic and hypercoagulable state, resulting from excessive levels of thyroid hormones that lead to systemic decompensation. The estimated incidence of thyroid storm among patients hospitalized for thyrotoxicosis ranges from 2% to 16%, with an overall mortality rate reported between 8% and 30%.^5^, ^6^, ^7^, ^8^, ^9^, ^10^, ^11^ The cardiovascular system is particularly affected, often presenting with increased cardiac output, tachycardia, and a heightened risk of arrhythmias, such as atrial fibrillation.^6^^,^^12^^,^^13^ The hypercoagulable state significantly elevates the likelihood of thrombus formation and subsequent ischemic events. In a study involving 64 hyperthyroid patients, 6.3% experienced thrombotic events, including myocardial infarction, cerebrovascular infarction, and pulmonary embolism.^14^ In the literature, some severe cases were described where myocardial infarction or cardiac arrest were the first symptoms of thyrotoxic crisis,^15^^,^^16^ underscoring the critical nature of early detection and intervention in these patients.

As described in this case, patients may also present with symptoms such as abdominal pain, nausea, vomiting, and diarrhea, which can easily be mistaken for other gastrointestinal or systemic conditions.^1^, ^2^, ^3^ The clinical manifestation of mesenteric ischemia in the setting of thyrotoxic storm is particularly complex and often nonspecific, which can result in the delay of the correct diagnosis. Looking at the timeline of events, the embolectomy and laparotomy were performed within 4 h after admission while the decision to proceed with thyroidectomy was made after 43 h. For patients with a known history of hyperthyroidism, thyrotoxic crisis is a clinical diagnosis.^11^^,^^17^^,^^18^ In this case, the patient initially denied any pre-existing medical conditions; however, her family later revealed a history of intermittent dyspnea lasting approximately 1 year. Additionally, she had experienced hot flashes for the past few weeks and diarrhea for the last 4 days. Her behavior was characterized as hyperactive, nervous, and occasionally agitated and restless. These symptoms may indicate underlying thyroid issues that may have persisted for some time. This information ultimately raised suspicion for a thyroid disease as the potential underlying cause of her condition.

Ennab et al described a similar case in which the patient was successfully treated with endovascular intervention of the SMA only. However, the patient presented with much milder symptoms, and open surgical management was not necessary. Additionally the patient had no peritoneal signs, was hemodynamically stable throughout and could quickly be discharged, continuing medical treatment with warfarin, Carbimazole, and propranolol.^4^

In the presented case, the patient did not stabilize after the embolectomy or the initial laparotomy with bowel resection. Burch and Wartowsky developed a point system that evaluates the extent of dysfunction in thyrotoxic crisis, with scores over 45 indicating thyroid storm.^7^ After a comprehensive thyroid evaluation, the presented patient had a score of 75, indicating a severe state. When investigating outcomes in patients who underwent uncommon urgent thyroidectomy, Song et al found that for patients with severe thyrotoxicosis experiencing life-threatening comorbidities, an urgent thyroidectomy offers a rapid and safe pathway to achieving clinical stability.^13^ Accordingly, in the presented case, it was only after urgent thyroidectomy that the patient stabilized.

In addition to the points discussed, it is important to consider that the thyroid storm may have been a consequence of the acute abdomen due to untreated hyperthyroidism. The presence of untreated hyperthyroidism can indeed increase the risk of thromboembolic events, potentially leading to complications such as SMA embolism like in our case.^14^^,^^19^ Additionally, the thyroid function tests were obtained 10 h after the embolectomy, which could also lead to further irregularities in the thyroid test results due to administration of iodinated contrast.^20^ If we were to account for this perspective, the Burch-Wartofsky Score could be adjusted upward by 10 points, resulting in a score of 85, which still indicates a high likelihood of thyroid storm. In both scenarios, whether the thyroid storm precipitated the acute abdomen or vice versa, an urgent thyroidectomy would remain indicated to address the underlying hyperthyroid state and stabilize the patient effectively.

In conclusion, this case highlights the rare but serious complication of mesenteric ischemia due to thyroid storm. The patient was successfully managed through a multidisciplinary approach involving endovascular thrombectomy, bowel resection, and thyroidectomy. This combination of treatments effectively resolved the acute vascular emergency while simultaneously stabilizing the underlying thyrotoxicosis. The case underscores the importance of early recognition and prompt intervention in thyroid storm-induced mesenteric ischemia, as well as the value of a comprehensive treatment strategy to improve outcomes. Endovascular thrombectomy and bowel resection effectively managed the ischemia, while thyroidectomy facilitated a rapid return to an euthyroid state, preventing further complications. This case serves as a valuable reference for managing similar complex presentations, demonstrating the potential for successful outcomes with timely and aggressive treatment.

## Disclosure

The authors have no conflicts of interest to disclose.
